# Supplementary material for: End Sequence Analysis Toolkit (ESAT) expands the extractable information from single-cell RNA-seq data
Source: Genome Res. 2016 Oct;26(10):1397–410. doi: 10.1101/gr.207902.116 (PMC5052061; doi:10.1101/gr.207902.116)
Supplement: Supplemental Material [file supp_gr.207902.116_Supp_Materials.tar.gz › ESAT-esat_v0.1/doc/html/classbroad_1_1pda_1_1seq_1_1protection_1_1_window_scores.html]

ESAT: broad.pda.seq.protection.WindowScores Class Reference


ESAT


- Main Page
- Related Pages
- Packages
- Classes
- Files

- Class List
- Class Index
- Class Hierarchy
- Class Members

All Classes Namespaces Files Functions Variables Enumerator Pages

- broad
- pda
- seq
- protection
- WindowScores

Protected Attributes |
Static Protected Attributes |
List of all members

broad.pda.seq.protection.WindowScores Class Reference

Collaboration diagram for broad.pda.seq.protection.WindowScores:

[legend]

|  |  |
| --- | --- |
| Protected Attributes | |
| SampleData | sample |
|  | |
| SampleData | ctrl |
|  | |
| Map< Gene, CountScore > | geneScores |
|  | |
| Map< Gene, Double > | geneAvgCoverage |
|  | |
| Map< Gene, Map< Annotation,   CountScore > > | windowScores |
|  | |
| int | windowSize |
|  | |
| int | stepSize |
|  | |
| Map< String, Collection< Gene > > | genesByChr |
|  | |
| Map< String, Gene > | genesByName |
|  | |
| double | expressionCutoffValue |
|  | |
| boolean | expressionByPval |
|  | |
| boolean | fullyContainedReads |
|  | |

|  |  |
| --- | --- |
| Static Protected Attributes | |
| static Logger | logger = Logger.getLogger(SampleData.class.getName()) |
|  | |

## Member Data Documentation

SampleData broad.pda.seq.protection.WindowScores.ctrl

protected

boolean broad.pda.seq.protection.WindowScores.expressionByPval

protected

double broad.pda.seq.protection.WindowScores.expressionCutoffValue

protected

boolean broad.pda.seq.protection.WindowScores.fullyContainedReads

protected

Map<Gene, Double> broad.pda.seq.protection.WindowScores.geneAvgCoverage

protected

Map<String, Collection<Gene> > broad.pda.seq.protection.WindowScores.genesByChr

protected

Map<String, Gene> broad.pda.seq.protection.WindowScores.genesByName

protected

Map<Gene, CountScore> broad.pda.seq.protection.WindowScores.geneScores

protected

Logger broad.pda.seq.protection.WindowScores.logger = Logger.getLogger(SampleData.class.getName())

staticprotected

SampleData broad.pda.seq.protection.WindowScores.sample

protected

int broad.pda.seq.protection.WindowScores.stepSize

protected

Map<Gene, Map<Annotation, CountScore> > broad.pda.seq.protection.WindowScores.windowScores

protected

int broad.pda.seq.protection.WindowScores.windowSize

protected

---

The documentation for this class was generated from the following file:

- java/broad/pda/seq/protection/WindowScores.java


---

Generated on Fri Aug 15 2014 15:11:03 for ESAT by  

 1.8.7
